# Molecular Interaction of a Kinase Inhibitor Midostaurin with Anticancer Drug Targets, S100A8 and EGFR: Transcriptional Profiling and Molecular Docking Study for Kidney Cancer Therapeutics

**DOI:** 10.1371/journal.pone.0119765

**Published:** 2015-03-19

**Authors:** Zeenat Mirza, Hans-Juergen Schulten, Hasan Ma Farsi, Jaudah A. Al-Maghrabi, Mamdooh A. Gari, Adeel Ga Chaudhary, Adel M. Abuzenadah, Mohammed H. Al-Qahtani, Sajjad Karim

**Affiliations:** 1 King Fahd Medical Research Center, King Abdulaziz University, PO BOX 80216, Jeddah, 21589, Saudi Arabia; 2 Center of Excellence in Genomic Medicine Research, Faculty of Applied Medical Sciences, King Abdulaziz University, PO BOX 80216, Jeddah, 21589, Saudi Arabia; 3 Department of Urology, Faculty of Medicine, King Abdulaziz University Hospital, Jeddah, Saudi Arabia; 4 Department of Pathology, Faculty of Medicine, King Abdulaziz University Hospital, Jeddah, Saudi Arabia; 5 Department of Pathology, King Faisal Specialist Hospital and Research Center, Jeddah, Saudi Arabia; 6 KACST Technology Innovation Center in Personalized Medicine at King Abdulaziz University, Jeddah, Saudi Arabia; Charles R. Drew University of Medicine and Science, UNITED STATES

## Abstract

The S100A8 and epidermal growth factor receptor (EGFR) proteins are proto-oncogenes that are strongly expressed in a number of cancer types. EGFR promotes cellular proliferation, differentiation, migration and survival by activating molecular pathways. Involvement of proinflammatory S100A8 in tumor cell differentiation and progression is largely unclear and not studied in kidney cancer (KC). S100A8 and EGFR are potential therapeutic biomarkers and anticancer drug targets for KC. In this study, we explored molecular mechanisms of interaction profiles of both molecules with potential anticancer drugs. We undertook transcriptional profiling in Saudi KCs using Affymetrix HuGene 1.0 ST arrays. We identified 1478 significantly expressed genes, including S100A8 and EGFR overexpression, using cut-off p value <0.05 and fold change ≥2. Additionally, we compared and confirmed our findings with expression data available at NCBI’s GEO database. A significant number of genes associated with cancer showed involvement in cell cycle progression, DNA repair, tumor morphology, tissue development, and cell survival. Atherosclerosis signaling, leukocyte extravasation signaling, notch signaling, and IL-12 signaling were the most significantly disrupted signaling pathways. The present study provides an initial transcriptional profiling of Saudi KC patients. Our analysis suggests distinct transcriptomic signatures and pathways underlying molecular mechanisms of KC progression. Molecular docking analysis revealed that the kinase inhibitor "midostaurin" has amongst the selected drug targets, the best ligand properties to S100A8 and EGFR, with the implication that its binding inhibits downstream signaling in KC. This is the first structure-based docking study for the selected protein targets and anticancer drug, and the results indicate S100A8 and EGFR as attractive anticancer targets and midostaurin with effective drug properties for therapeutic intervention in KC.

## Introduction

Cancer is a global major health problem. Dysregulation in molecular signaling pathways is a hallmark of cancer initiation and progression [1–3]. Kidney cancer (KC) accounts for approximately 1.5 percent of all cancer deaths. In particular, KC is common in obese male population [4]. Surgical tumor resection is the standard curative treatment. Metastatic KC is almost nonresponsive to conventional systemic treatments and nearly all patients die of metastasis. Lack of promising biomarkers for effective targeted chemotherapy poses a big challenge in KC management. Better understanding of the molecular mechanisms effective in KC have widened the window for development of effective targeted therapies [5,6]. High-throughput microarray platforms are well suited for identification of the novel induced or suppressed disease-related culprit genes [7]. Molecules showing direct involvement in a biochemical or regulatory pathway leading to disease are potential anticancer target. Drug/molecule interaction involving these targets can either be investigated by co-crystallization or tested by docking simulation to indentify molecular interactions required for rational drug designing [8]. High-throughput docking is the key entrance for drug discovery [9,10]. Transcriptomic profiling and functional pathway analysis in KC have identified several significantly differentially expressed genes, including S100A8 and EGFR. We tried to evaluate their potential as KC drug target by *in silico* docking with known protein kinase inhibitors. Overall, this study illustrates structure-based virtual screening and ligand-protein docking of anticancer drugs e.g. midostaurin, enzastaurin, and gefitinib, with anticancer targets, S100A8 and EGFR.

S100A8 is a small (10 kDa) proinflammatory protein of S100 family, which tends to form heterodimeric complexes with S100A9 (S100A8/A9) [9], that undergo conformational changes upon Ca^2+^ binding and function as intracellular Ca^2+^ sensors [10]. Under physiological conditions, these Ca^2+^ binding EF hand type proteins are constitutively expressed by myeloid cells [11–13]. However, under pathological conditions like inflammation and cancer, an increased expression of S100A8 is seen in epithelial cells [14,15]. Early stage death of S100A8 knock-out mice proves the essentiality of this gene for survival [16]. Enhanced level of S100A8 is found in different carcinomas including breast [15], prostate [17,18], lung [19], gastric [20], hepatic [21], pancreatic [22] and colorectal cancer [23,24]. A recent study shows cell growth-promoting activity and binding to receptor for advanced glycation endproducts (RAGE) at low S100A8 concentrations [15]; however, its direct role in tumorogenesis is ambiguous and has to be elucidated yet. It has been reported that primary tumors secrete soluble factors, which induce expression of S100A8 in the endothelial cells prior to tumor metastasis [19]. By activating the p38 MAPK pathway, it increases the motility of circulating cancer cells [25]. Therefore targeting S100A8 could be used to prevent the tumor cell migration and growth. Several lines of evidence point to vital functions of S100A8 during tumorigenesis and, although it’s exact role within the tumor microenvironment is still not clear, different tumor-promoting effects have been proposed. Though, research is limited on its expression pattern in cancer, its involvement in oncogenesis and its potential as therapeutic biomarker. To the best of our knowledge, no study has reported yet its expression pattern or its role in KC progression.

EGFR is one of the four member of the ErbB family of receptor tyrosine kinases. The receptor is overexpressed or mutated in many cancers, highlighting its role as therapeutic cancer biomarker. It is involved in diverse cellular functions such as proliferation, angiogenesis and suppression of cell death. Being a transmembrane protein, EGFR passes crucial signals from epithelial cell surface to the intracellular domain for controlled cell proliferation, migration and adhesion. Overexpressed EGFR transmits multiple signals to cell for accelerated growth and cellular longevity, and plays a key role in the carcinogenesis of different types of cancer [26–29]. Improved perception of the molecular signaling pathways has opened new strategies and ways for cancer treatment. Thus, targeting EGFR to turn off its signal transduction is envisaged to block growth and survival of cancer cells.

S100A8 and EGFR do play an important role in malignancy and thus considered as potential drug targets for cancer therapeutics development [26–30]. The reason for choosing midostaurin, enzastaurin, and gefitinib as inhibitor for S100A8 and EGFR is the recent research showing that EGFR-targeted therapies using kinase inhibitors are effective in many cancer patients [31–36]. Gefitinib, an inhibitor of EGFR kinase function, was approved by the U.S. FDA for lung cancer treatment [37]. S100A8 does not own any kinase domain or ATP binding pocket, however it participates in protein phosphorylation [38] and is an upstream pathway molecule of EGFR. Thus, a drug capable of inhibiting both EGFR and S100A bears prospective therapeutic impact.

After the discovery of protein kinase activity in 1954, a lot of kinase inhibitors have been identified [39]. Because of high degree of similarity in their structure and function within the “kinome” [40], identification of effective protein kinase inhibitors (PKIs) is a great challenge. At present kinase inhibitors comprise more than 30% of drug-discovery programs resulting in approval of dozens of kinase inhibitors as anti-cancer drugs or at least testing in clinical trials [41]. In this study, we determined by docking simulation the potential efficiency of three such drugs named midostaurin, enzastaurin, and gefitinib.

(i) Midostaurin (CID 104937; also known as PKC412 and benzoylstaurosporine) is a multi-target kinase inhibitor used for acute myeloid leukemia treatment. It is a semi-synthetic alkaloid derived from bacterial staurosporine used to treat patients with CD135 (FMS-like tyrosine kinase 3 receptor) mutations [42]. Midostaurin inhibits growth or induces apoptosis in several types of cancer, blocks angiogenesis and sensitizes cancer cells to ionizing radiation, justifying its use in cancer treatment [43]. Preclinical studies have shown that midostaurin works synergistically with chemotherapy agents, reinforcing each other's effect against cancer [44]. (ii) Enzastaurin is a synthetic acyclic bisindolylmaleimide showing antineoplastic activity. It is a small oral serine/threonine kinase inhibitor of PKCβ and AKT pathways. It inhibits protein kinase Cβ that is known stimulator of neo-angiogenesis through induction of vascular endothelial growth factor (VEGF). Binding of protein kinase Cβ to the ATP-binding site of VEGF probably decreases tumor blood supply and prevents growth. Apart from its anti—VEGF factor effects, low concentration of enzastaurin suppresses proliferation in cancer cell lines [45]. Loss of GSK3β and AKT phosphorylation has been reported in myeloma cells treatment with enzastaurin [46]. (iii) Gefitinib (Iressa) selectively targets the ATP cleft within EGFR tyrosine kinase domain [47]. It interrupts EGFR signaling of target cells and therefore is effective only in cancer cells with mutated and overactive EGFR.

Molecular docking forecasts an optimized conformation and relative orientation for both the protein and ligand molecule. In this study we were aiming to evaluate the potential of S100A8 and EGFR as KC drug target by *in silico* molecular docking with the known protein kinase inhibitors midostaurin, enzastaurin, and gefitinib to test the effectiveness of these anticancer drugs under these constellations.

## Materials and Methods

### Patients and samples

The study was performed on patients from Saudi Arabia diagnosed with renal cell carcinoma. The samples were collected from King Abdulaziz University Hospital, Bakhsh Hospital and King Faisal Specialist Hospital & Research Center, Jeddah during 2010–2012. For gene expression analysis, fresh tumor and normal tissue specimens were taken from surgical resections of tumor and normal kidney tissues, respectively and were immediately placed in RNALater (Invitrogen—Life Technologies, Grand Island, NY, USA). Out of 18 tumor specimen, only two passed the criteria (RNA integrity number (RIN) >5) to be used for array expression analysis. One patient was 61 year old Saudi male, diagnosed with clear cell renal cell carcinoma of nuclear grade II and tumor size 4.5 x 3 x 4 cm. The second patient was 47 year old Saudi female, diagnosed with chromophobe renal cell carcinoma of Fuhrman’s grade II.

#### Ethical approval

All patients included in the study provided written informed consent. The study was reviewed and approved by the Center of Excellence in Genomic Medicine Research (CEGMR) local ethical committee (approval number 08-CEGMR-02-ETH).

### RNA extraction and array processing

Total RNA was extracted from freshly preserved kidney tissue specimens with the Qiagen RNeasy Mini Kit (Qiagen, Hilden, Germany) including an on-column DNAse treatment according to manufacturer’s recommendations. Quality of the purified RNA was verified on an Agilent 2100 Bioanalyzer (Agilent Technologies, Palo Alto, CA). Mean value of RIN for processed samples was 8.0 only for two cancer samples and four normal kidney tissues. RNA concentrations were determined using a NanoDrop ND-1000 spectrophotometer (NanoDrop Technologies, Wilmington, DE). RNA samples (250 ng) were processed according to the manufacturer’s recommendations (Life Technology, Grand Island, NY). After fragmentation and labeling, the samples were hybridized at 45°C for 17 hours to Human Gene 1.0 ST GeneChip arrays (Affymetrix, Santa Clara, CA, USA). These arrays are conceptually based on the Human Genome sequence assembly UCSC hg18, NCBI Build 36 and interrogated with a set of 764,885 probes 28,869 annotated genes.

### Gene Expression Analysis

We conducted expression profiling of two renal cell carcinomas and four normal kidney tissues. Because of our limited number of samples, independent datasets available in public domain were incorporated in the gene expression analysis process. We used selected KC expression data from NCBI's GEO database (Accession no: GSE781, GSE7023, and GSE6344) for comparative analysis and confirmation. Sample size for GSE781, GSE7023, and GSE6344 were 34, 47, and 40 respectively. Affymetrix. CEL files were imported to Partek Genomics Suite version 6.6 (Partek Inc., MO, USA). The data was normalized using RMA normalization. Analysis of Variance (ANOVA) was applied on the complete data set and the list of differentially expressed genes was then generated using false discovery rate (FDR) of 0.05 with 2-fold change cut off. Unsupervised two-dimensional average linkage hierarchical clustering was performed using Spearman’s correlation as a similarity matrix.

### Functional and Pathway analysis

To define biological networks, interaction and functional analysis among the differentially regulated genes in KC, pathway analyses were performed using Ingenuity Pathways Analysis software (IPA) (Ingenuity Systems, Redwood City, CA). Statistically differentially expressed datasets with probesets ID, Gene symbol and Entrez gene ID as clone identifier, p-value and fold change values were uploaded into IPA. The functional/pathway analysis of IPA identifies the biological functions and/or diseases and pathways that are most significantly altered for the differentially expressed gene set. The significance of the connection between the expression data and the canonical pathways were calculated by ratio and/or Fisher’s exact test.

### Molecular Docking

**Fig 1:**
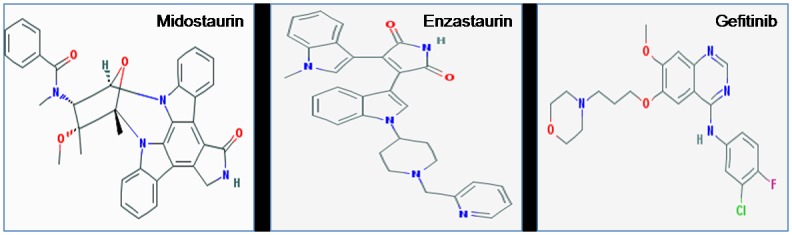
Two dimensional molecular structure of the three anti-cancer drugs: midostaurin, enzastaurin and gefitinib.

**Fig 2:**
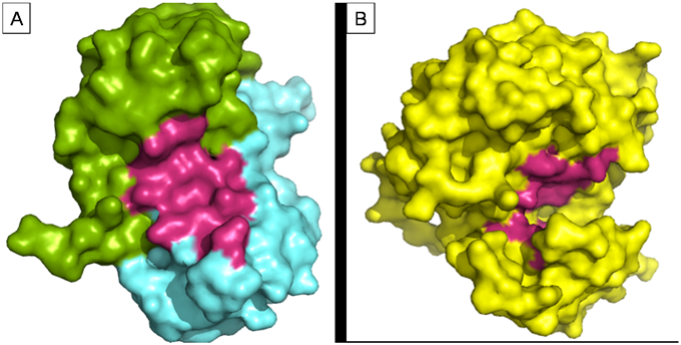
Structure visualization of cancer signaling target proteins S100A8 (1MR8), and EGFR tyrosine kinase domain (2GS2) retrieved from Protein Data Bank. Surface representation of the two PDB structures used for docking analysis. Figure made using PyMol. (1) MR8 chain A (green) + chain B (cyan) (2) 2GS2 chain (yellow), drug binding cavity in magenta.

We performed molecular docking studies (i) to investigate the role of S100A8 as inflammatory mediators and to establish their involvement in inflammation-associated cancers at molecular level and (ii) to investigate the role of EGFR signaling in proliferation, angiogenesis and suppression of cell death causing cancer. The 3-D structures of identified cancer drug targets were retrieved from Protein Data Bank (PDB ID: S100A8 dimer, 1MR8 and EGFR tyrosine kinase domain, 2GS2). The molecular structures of inhibitors were retrieved from PubChem compound database (midostaurin with CID 104937; enzastaurin with CID 176167 and gefitinib with CID 123631) (Fig. 1). The bound ligand was used as probe for the binding site grid generation. Structure visualization and identification of drug binding site was done using PyMol [48] (Fig. 2). PyMOL was used to analyze and generate an illustration of whole protein-ligand complex.

Docking calculations were done with Molecular Docking Server [49]. Merck molecular force field 94 (MMFF94) [50] was used for energy minimization of drug molecules used as ligand: midostaurin, enzastaurin and gefitinib. Gasteiger partial charges were added to ligand atoms. Non-polar hydrogen atoms were combined, and rotatable bonds were defined. Molecular docking of each ligand was performed individually with (S100A8)_2_ homo-dimmer (1MR8), and EGFR tyrosine kinase domain (2GS2) protein models, to predict the binding orientation and interaction. Essential hydrogen atoms, Kollman united atom type charges, and solvation parameters were added with the aid of AutoDock tools [51]. Autogrid program was used to generate affinity (grid) maps of 20×20×20 Å grid points and 0.375 Å binding site grid generation spacing [52]. AutoDock parameter set- and distance-dependent dielectric functions were used in van der Waals and the electrostatic terms calculation, respectively. Docking simulations were achieved using the Lamarckian genetic algorithm (LGA) and the Solis & Wets local search method [53]. Orientation, initial position, and torsion angles of ligand molecules were set randomly. Each docking experiment was the resultant of 10 different runs that were set to cease after a maximum of 250000 energy evaluations. Population size was set to 150. During the search, a translational step of 0.2 Å, and quaternion and torsion steps of 5 were applied in the current series of docking analysis.

### Availability of supporting data

The data sets supporting the results of this study are available in the NCBI's Gene Expression Omnibus (GEO), identified as GSE781, GSE7023, and GSE6344 (http://www.ncbi.nlm.nih.gov/gds/?term=GSE781).

## Results

The main focus of this study was to discover novel anticancer drug target by transcriptomic profiling and to identify possible protein-drug interactions by molecular docking analysis. We identified S100A8 and EGFR as important proteins of KC and made an attempt to demonstrate their anticancer drug target potential.

### Identification of differentially expressed genes

**Fig 3:**
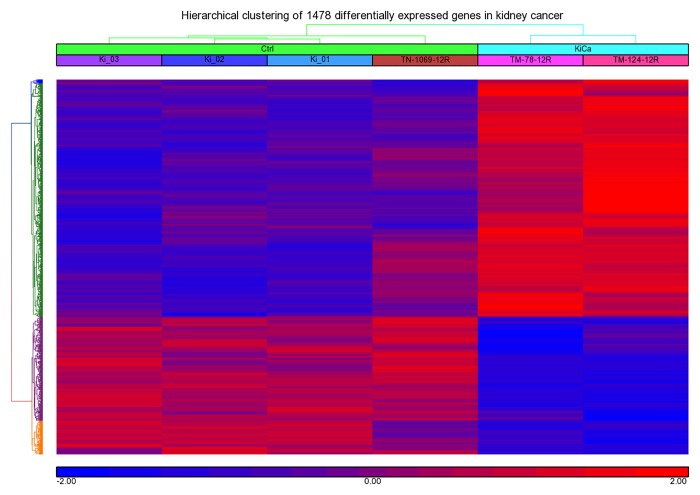
Hierarchical clustering and functional analysis of significantly differentially expressed genes in kidney cancer using Affymetrix Human ST 1.0 array and Partek Genomics suite (ver 6.6).

We profiled fresh kidney tissue specimens and compared them with normal control samples. Clear differences were also observed between the tumors and normal tissues revealing distinct expression profiles for each tissue types. Comparison of the genome-wide expression of KC revealed 1478 differentially expressed genes, 943 up-regulated and 535 down-regulated, with a ≥ 2 fold change and false discovery rate of p < 0.05 (Fig. 3, Table 1, S1 Table). Identified differentially expressed genes in our dataset were compared with re-analyzed dataset (GSE-781, -6344 and-7023) retrieved from GEO database. 876, 1200 and 1258 differentially expressed genes were found significant for GSE781, GSE6344 and GSE7023 dataset respectively at cut off value of fold change >2 and p value <0.05. As expected, there were hundreds of genes showing similar expression pattern including S100A8 and EGFR, both gene were over-expressed in all dataset under study except GSE7023, where fold change were 1.948 for S100A8 and 1.893 for EGFR. Elevated expression of S100A8 and EGFR in our CEGMR dataset and GEO dataset is confirming our finding (Table 2, S2 Table).

**Table 1:** Differentially expressed significant genes in Kidney cancer.

Gene Symbol	Gene_assignment	Transcript ID	RefSeq	p-value	Fold-Change
TOPBP1	topoisomerase (DNA) II binding protein 1	8090772	NM_007027	1.11E-06	2.05612
TDO2	tryptophan 2,3-dioxygenase	8097991	NM_005651	7.91E-06	3.30054
FOXM1	forkhead box M1	7960340	NM_202002	8.08E-06	2.89359
NPHS2	nephrosis 2, idiopathic, steroid-resistant (podocin)	7922627	NM_014625	1.56E-05	-41.0848
C3orf58	chromosome 3 open reading frame 58	8083223	NM_173552	1.65E-05	2.13922
UMOD	uromodulin	7999936	NM_003361	1.66E-05	-150.524
ANKRD13A	ankyrin repeat domain 13A	7958600	NM_033121	3.83E-05	2.78943
KCNJ1	potassium inwardly-rectifying channel, subfamily J, member 1	7952617	NM_153767	4.93E-05	-24.6069
ANKRD2	ankyrin repeat domain 2 (stretch responsive muscle)	7929653	NM_020349	4.95E-05	-3.11353
CALB1	calbindin 1, 28kDa	8151730	NM_004929	5.79E-05	-158.598
PRSS42	protease, serine, 42	8086683	NM_182702	5.84E-05	-2.01485
GLTPD2	glycolipid transfer protein domain containing 2	8003948	NM_001014985	5.90E-05	-2.68064
ESCO1	establishment of cohesion 1 homolog 1 (S. cerevisiae)	8022473	NM_052911	5.99E-05	2.10289
NTRK2	neurotrophic tyrosine kinase, receptor, type 2	8156134	NM_006180	6.46E-05	-2.76027
BLM	Bloom syndrome, RecQ helicase-like	7986068	NM_000057	7.64E-05	2.14272
SLC12A3	solute carrier family 12 (sodium/chloride transporters), member	7995868	NM_000339	8.17E-05	-61.4283
ITGA6	integrin, alpha 6	8046380	NM_000210	8.51E-05	2.82412
SPN	sialophorin	7994603	NM_001030288	0.00010918	2.01423
S100A8	S100 calcium binding protein A8	7920244	NM_002964	0.0159132	2.66364
EGFR	epidermal growth factor receptor (erythroblastic leukemia viral (v)	8132860	NM_005228	0.0410945	3.40122

Negative fold change value indicates the downregulation.

**Table 2:** Expression of S100A8 and EGFR in kidney cancer among Saudi patients (CEGMR dataset) and GEO dataset (GSE781, GSE6344 and GSE7023).

Gene Symbol	CEGMR (own data) Sample size = 6		GSE781 Sample size = 34		GSE6344 Sample size = 40		GSE7023 Sample size = 47	
	Fold Change	p-value	Fold Change	p-value	Fold Change	p-value	Fold Change	p-value
S100A8	2.663	0.0159	2.759	0.0109	3.107	0.0019	1.948	0.0105
EGFR	3.401	0.0410	5.563	8.54E-05	3.32472	6.00E-08	1.893	0.0026

### Pathways and networks underlying kidney cancer

**Fig 4:**
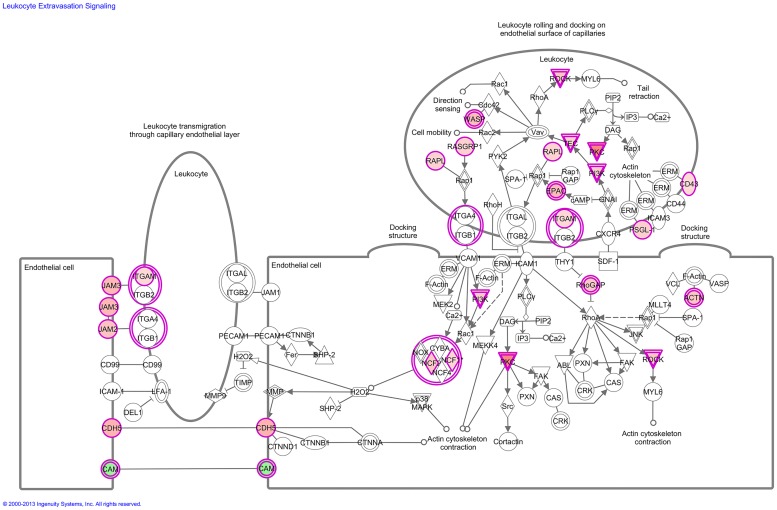
Leukocyte Extravasation Signaling: Transcriptomic signatures of kidney cancer showed a significant activation in leukocyte extravasation signaling pathway. Red represents overexpression and green underexpression.

To understand the mechanisms by which the genes alter a wide range of physiological processes, we examined biofunctions, molecular network and pathways associated with KC. Interestingly, the biological process, cellular movement was significantly overrepresented in both down-regulated and up-regulated gene lists pointing that the metastasis is probably linked to a different equilibrium of switching on and off. Functional analysis of KC-associated genes found an over expression of genes involved in cell cycle progression, DNA repair, cell death, tumor morphology and tissue developments. Pathway analysis showed significant disruption in certain signaling pathways including atherosclerosis signaling, LXR/RXR activation, leukocyte extravasation signaling, hepatic fibrosis / hepatic stellate cell activation, notch signaling, and IL-12 signaling and production in macrophages (Fig. 4, Table 3). Extensive pathway analysis of differentially regulated genes may provide novel hypotheses underlying tumor invasion and metastatic progression of KC.

**Table 3:** Canonical pathways predicted by Ingenuity Pathway Analysis for significant genes differentially expressed in kidney cancer.

Ingenuity Canonical Pathways	-log (p-value)	Down-regulated	Upregulated	Molecules
**Atherosclerosis Signaling**	3.14E00	5/136 (4%)	13/136 (10%)	APOE,APOM,MSR1,PLA2R1,PLA2G7,SELPLG,COL1A2,APOC1,APOL1,COL1A1,IL18,ALB, LYZ,CCL2, S100A8,PDGFD,RBP4,COL3A1
**LXR/RXR Activation**	2.93E00	8/139 (6%)	10/139 (7%)	KNG1,SCD,APOE,APOM,ECHS1,MSR1,AMBP,ABCG1,APOC1,APOL1,IL18,ALB,LYZ, LY96, CCL2, S100A8,HADH,RBP4
**Leukocyte Extravasation Signaling**	2.65E00	4/205 (2%)	20/205 (10%)	CLDN10,ARHGAP6,SPN,PIK3C2A,CLDN19,JAM2,ITGA6,RAPGEF4,SELPLG,PIK3R3,BTK,ROCK1,NCF1,WIPF1,ITGAM,CDH5,JAM3, CLDN16,RASGRP1,NCF2,PRKCH,RASSF5, ACTN1,CLDN3
**Hepatic Fibrosis / Hepatic Stellate Cell Activation**	2.52E00	3/153 (2%)	15/153 (10%)	FN1,CXCL9,FGFR1,EGF,FGF1,BCL2,COL1A2,COL1A1,LY96,IGF1,CCL2,TGFB2,IL10RA, MYH9, EDNRA,ECE1,MYL3,COL3A1
**Maturity Onset Diabetes of Young (MODY) Signaling**	2.46E00	5/29 (17%)	1/29 (3%)	HNF1B,PKLR,ALDOB,SLC2A2,CACNA1C, FABP1
**Coagulation System**	2.21E00	5/38 (13%)	2/38 (5%)	F11,KNG1,PLG,SERPINA5,PROC,VWF, PLAUR
**Valine Degradation I**	2.21E00	6/35 (17%)	1/35 (3%)	HIBCH,BCAT1,ECHS1,ABAT,ACADSB,EHHADH,ALDH6A1
**Notch Signaling**	2.07E00	1/42 (2%)	6/42 (14%)	DLL1,ADAM17,DTX1,JAG2,MAML3,DLL4, HEY1
**Intrinsic Prothrombin Activation Pathway**	2.03E00	3/36 (8%)	3/36 (8%)	F11,KNG1,COL1A2,COL1A1,PROC,COL3A1
**IL-12 Signaling and Production in Macrophages**	1.88E00	5/154 (3%)	11/154 (7%)	PPARG,APOE,APOM,PIK3C2A,MST1,APOL1,PIK3R3,APOC1,ALB,LYZ,IL18,TGFB2,MAP3K8, S100A8,PRKCH,RBP4

### Docking studies

**Fig 5:**
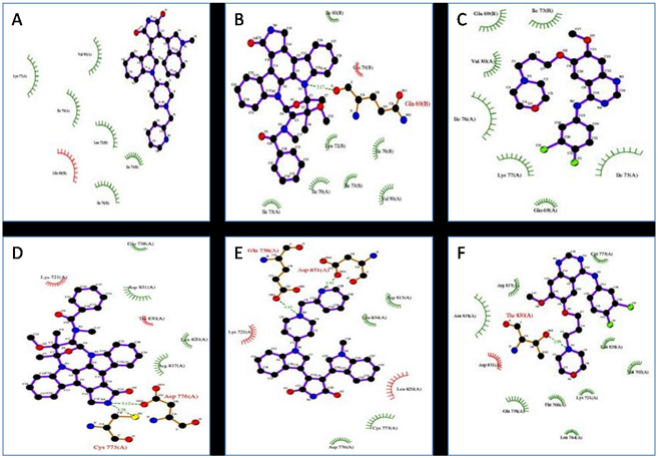
2D plot of inhibitors with S100A8 and EGFR tyrosine kinase domain proteins interaction profile by DockingServer. Ligand bond, non-ligand bond, hydrogen bond and their lengths are marked for midostaurin, enzastaurin and gefitinib. Where A, B, C shows interaction of S100A8 (1MR8) with the drugs, and E, F shows interaction of EGFR (2GS2) with midostaurin and gefitinib respectively.

**Fig 6:**
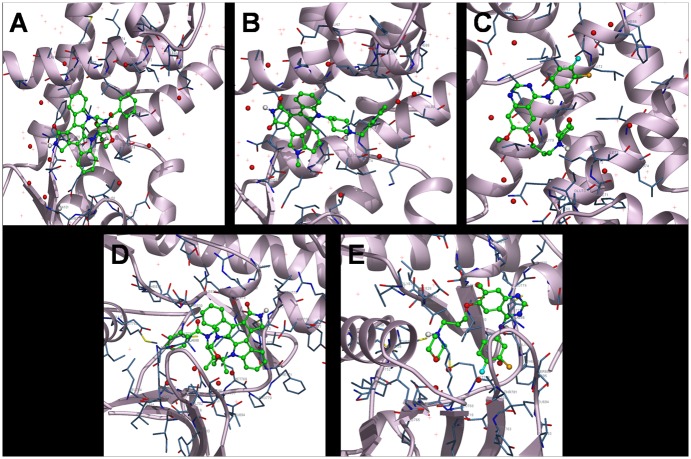
Interactions of ligand with the protein. Red represents protein as cartoon; grey represents interacting side chain as cylinder; and green represents drug as ball and stick model.

Molecular docking studies predicted potential interactions of our proposed protein drug target with the selected drug molecules. This is a structural modeling approach to study possible binding for cancer therapeutics. To understand the molecular interaction between drugs and S100A8, series of molecular docking analysis were performed using three dimensional structure available (PDBID: 1MR8) with three anti-cancer drugs i.e. midostaurin, enzastaurin and gefitinib. The ligand binding site was a hinge region containing two EF-hand motifs. In similar way, we also simulated docking of the above-mentioned drugs with the tyrosine kinase domain of EGFR. Based on their size, stereochemistry and structural differences the ligands exhibited varied intensity in binding with the protein target molecules. The predicted parameters of estimated binding free energy, inhibition constant (Ki), total energy of vdW + Hbond + desolvation + electrostatic energy, total intermolecular energy and interacting surface area were evaluated to estimate the favorable binding of ligand drug molecules to the target protein. Molecular visualization was performed using PyMol. Complete interaction profile (H-bonds, polar, hydrophobic, pi-pi, cation-pi and other contacts), and hydrogen bonding (HB plot) interactions were studied (Figs. 5 and 6, Table 4).

**Table 4:** Binding and interaction values for docking of S100A8 dimer and EGFR kinase domain with inhibitors (midostaurin, enzastaurin and gefitinib).

(S100A8)_2_ (1MR8)	Midostaurin	Enzastaurin	Gefitinib
Est. Free Energy of Binding	-9.77 kcal/mol	-4.19 kcal/mol	-4.21 kcal/mol
Est. Inhibition Constant, Ki	68.48 nM	844.18 μM	813.89 μM
vdW + Hbond + desolv Energy	-7.65 kcal/mol	-6.33 kcal/mol	-6.11 kcal/mol
Electrostatic Energy	-0.03 kcal/mol	-0.02 kcal/mol	-0.07 kcal/mol
Total Intermolec. Energy	-7.68 kcal/mol	-6.35 kcal/mol	-6.18 kcal/mol
Interact. Surface	754.992	849.622	667.469
**EGFR kinase domain (2GS2)**	**Midostaurin**	**Enzastaurin**	**Gefitinib**
Est. Free Energy of Binding	-6.58 kcal/mol	+7.10 kcal/mol	-4.15 kcal/mol
Est. Inhibition Constant, Ki	15.13 μM	-----	905.30 μM
vdW + Hbond + desolv Energy	-1.49 kcal/mol	+0.44 kcal/mol	-6.07 kcal/mol
Electrostatic Energy	-0.16 kcal/mol	-0.88 kcal/mol	-0.17 kcal/mol
Total Intermolec. Energy	-1.65 kcal/mol	-0.44 kcal/mol	-6.24 kcal/mol
Interact. Surface	911.021	895.39	795.106

The docking studies reveal the presence of one midostaurin molecule at the substrate-binding site of S100A8 dimer (PDB code 1MR8). It is observed that the drug molecule is buried in the substrate-binding hydrophobic channel of the S100A8 ligand binding domain. Most of the interacting residues are hydrophobic in nature. Interaction and accessible surface area analysis reveal that the cavity involved in the binding site of drugs has a molecular accessible surface area of 123.017 Å^2^ and a solvent accessible surface area of 509.834 Å^2^. The conformations of the binding site in S100A8 as well as that in drugs were not altered upon binding as rigid docking simulation was carried out. The binding cavity is the same for all three drugs.

We selected the crystal structure of the active EGFR kinase domain having a length of 330 residues (PDB code 2GS2) for docking study. The drug binding cavity of the EGFR tyrosine kinase domain is more like a channel and is deeper as compared to the S100A8 case and has a molecular and solvent accessible surface area of 181.409 Å^2^ and 875.519 Å^2^ respectively. The cavity is lined with mostly polar as well as few non-polar residues. Enzastaurin did not exhibit any binding with the selected tyrosine kinase domain. Gefitinib is a previously known inhibitor of EGFR and its co-crystal structure has been determined and presented in PDB (3UG2), however docking studies revealed that midostaurin is the more suitable binding partner of EGFR than gefitinib.

#### (S100A8)_2_ (1MR8) with midostaurin

Midostaurin binds at the ligand binding site and forms an H-bond with a critical amino acid residue in the ligand binding domain site of S100A8 i.e. Gln 69 (B) formed between N1 of ligand and the terminal O of Gln. The residues involved in ligand cavity formation are two polar residues Gln 69 (B) and Glu 70 (B) and other hydrophobic residues Ile 60 (B), Ile 73 (A), Ile 73 (B), Ile 76 (A), Ile 76 (B), Val 80 (A) and Leu 72 (B) as shown in Fig. 2. More than 20 hydrophobic contacts and van der Waals interactions are also noted. The inhibitor exhibits favorable binding characteristics with the predicted free binding energy-9.77 kcal/mol and estimated inhibition constant, Ki of 68.48 nM. These results are promising and are superior to other docking results done in this particular study.

#### (S100A8)_2_ (1MR8) with enzastaurin

The drug binds to the protein molecule satisfactorily with an estimated free binding energy of-4.19 kcal/mol and Ki of 844.18 μM. It shows binding with the S100A8 dimer but without the formation of neither any H-bonds nor polar contacts. It displays five hydrophobic contacts mediated by aliphatic non-polar amino acids Leu 72 (B), Ile 73 (B), Ile 76 (A), Ile 76 (B), and Val 80 (A). Other noticeable interactions are with Lys 77 (A) and Gln 69 (B).

#### (S100A8)_2_ (1MR8) with gefitinib

Binding ability of gefitinib is good but there are no H-bonds formed and the free energy of bound structure is-4.21 kcal/mol. One noticeable polar interaction is with Lys 77 of protein chain A. There are around ten hydrophobic interactions with the drug binding site lining hydrophobic residues—Ile 73 (A), Ile 73 (B), Ile 76 (A) and Val 80 (A). Halogen atoms F and Cl of the drug show non-covalent interaction. For protein-ligand complexes, halogen bonds are energetically and geometrically comparably equal to that of hydrogen bonding if the donor-acceptor directionality remains consistent. This intermolecular interaction has been shown to be stabilizing and a conformational determinant in complexes [54]. F displays a water mediated bond and Cl shows interaction with Gln 69 (A). A couple of other weak interactions was noticed too.

#### EGFR kinase domain (2GS2) with midostaurin

Midostaurin being primarily a kinase inhibitor docks well with the EGFR tyrosine kinase domain with free binding energy of-6.58 kcal/mol and inhibition constant of 15.13 μM. Four H-bonds are possibly involved between the protein and ligand molecule. Cys 773 Sγ shows H-bonding with N4 of drug at a interatomic distance of 3.28 Å. In addition, atom 0δ2 of Cys 773 likely forms another H-bond with H atom of ligand. Similar pattern of H-bond is seen with Asp 776. Residues Arg 817, Thr 830 and Asp 831 are involved in polar interactions with the ligand. Hydrophobic interactions are mediated by Cys 773 and Leu 820. Other interactions with the drug are mediated through Lys 721, Glu 738, Asp776, Arg 817, Leu 820, Thr 830 and Asp 831.

#### EGFR kinase domain (2GS2) with enzastaurin

The docked structure shows positive free energy of binding implying that binding is not feasible as most of the decomposed interaction energies have positive value. The result can be partly explained by the fact that enzastaurin is mainly a serine/threonine kinase inhibitor but we have docked it with EGFR tyrosine kinase domain. Otherwise, enhanced docking properties can be possibly achieved by increasing the simulation box size.

#### EGFR kinase domain (2GS2) with gefitinib

In our docking analysis total of three H-bonds are seen, two with Thr 830 (interatomic distance of 2.99 and 3.89 Å) and one with Glu 738 (3.48 Å). Glu 738, Asn 818 and Asp 831 display polar interactions. Leu 764, Cys 773 and Leu 820 exhibit hydrophobic interactions. Two water-mediated halogen bonds are seen with F. Several other interactions are noticed with Val 702, Lys 721, Glu 738, Thr 766, Cys 773, Arg 817, Asn 818, Leu 820, Thr 830 and Asp 831. The monomeric (V948R) gefitinib/erlotinib resistant double mutant (L858R+T790M) EGFR kinase domain has been previously co-crystallized with gefitinib (PDB ID: 4I22) [55].

## Discussion

Kidney cancer comprises heterogeneous tumors with diverse molecular and clinical characteristics as reflected by their response to specific treatments. To understand the mechanisms by which genes alter a wide range of physiological processes, we performed a transcriptional profiling study to identify significant genes and examined their biological functions, and to identify KC associated molecular network and pathways. Among hundreds of differentially expressed genes we identified S100A8 and EGFR as potential biomarker of KC and attempted to demonstrate their anticancer drug target potential. *In silico* docking analysis showed that midostaurin and gefitinib binds to S100A8 as well as to EGFR and predictably inhibits downstream signaling in KC. However, enzastaurin binds only to the S100A8 dimer. Our finding leads to the hypothesis that S100A8 and EGFR are promising anticancer drug targets and midostaurin can be a potential inhibitor. This hypothesis surely needs further validation.

S100 proteins participate in numerous functions including protein phosphorylation, enzymatic activation, calcium homeostasis, and interaction with cytoskeletal components [38]. Most genes encoding S100 proteins are clustered on a region of human chromosome 1q21 that is prone to chromosomal rearrangements, suggesting a link between S100A8/A9 proteins and metastasis and tumor formation [38,56]. S100A8 has cell growth-promoting activity at low concentrations by binding to RAGE. This binding enhances cell mesenchymal properties, induces epithelial-mesenchymal transition and plays important role in promoting cancer invasion and metastasis in cancer [15,22]. Abnormal expressions of S100A8 proteins were observed in a variety of cancers, such as gastric, lung, breast, liver, pancreatic and squamous esophageal carcinomas [15,17,20–23,57–60]. These studies indicate the potential of S100A8 as anticancer target. Despite showing the elevated expression and distinct role of S100A8 in different cancer types, less is known about the expression status or role of S100A8 in KC progression.

The EGFR family of RTPKs is an important cancer target because of the complex signaling through their configuration as homo- or hetrodimers [61,62]. The 4-anilino-quinazoline gefitinib (Iressa) targets the active conformation of EGFR kinase and has been approved for second- and third-line chemotherapy for advanced non small cell lung carcinoma (NSCLC) [63]. Interestingly, a subset of patients who have specific activating mutations in the EGFR tyrosine kinase domain and/or amplification of EGFR often display enhanced sensitivity, positive clinical responses and improved survival with these inhibitors [64,65]. However, the gatekeeper mutation T790M in EGFR enhances affinity for ATP and reduces affinity for gefitinib and thereby inducing resistance [66,67]. We, therefore, used another kinase inhibitor, midostaurin to determine their efficiencies. In our docking study, the best overall binding was exhibited in terms of estimated free energy of binding and Ki value by midostaurin followed by gefitinib and at least by enzastaurin. Interestingly, enzastaurin did not show any binding with EGFR. Enzastaurin has been previously evaluated both *in vitro* and in nude mice alone and in combination with the EGFR inhibitor gefitinib, where it showed a cooperative inhibitory effect with gefitinib in parental and in gefitinib-resistant cells [68].

This was a pioneering structure-based approach to study S100A8 and EGFR protein interactions with the chosen kinase inhibitors at the molecular level. The EGFR binding cavity is quite hydrophobic and is the most preferable binding site for compounds having compatible shape and stereochemistry. The computational results provide valuable insights into the binding modes of the three tested inhibitors to the S100A8 and EGFR targets. It has been demonstrated that the hydrophobic interactions and the hydrogen bonding with these targets have pivotal contributions to the binding structures and binding free energies, although the van der Waals and electrostatic interactions contributed to the stabilization of the binding structures. The calculated binding free energies comply with the available experimental activity data. The detailed structural insight, binding modes and the crucial factors affecting the binding free energies obtained from the present computational studies may provide valuable insights for future rational structure-based design of novel and potent inhibitors.

The size of a single amino acid in the ATP binding pocket—termed the gatekeeper residue—is a critical determinant of inhibitor sensitivity [67]. Kinases which possess a threonine at this position are sensitive to a range of inhibitors, whereas those having a larger amino acid side chain show significantly higher IC_50_ values than those having a threonine residue at this site and are broadly resistant [69]. For an ATP-competitive kinase inhibitor, a micromolar inhibition constant, Ki alone is compelling evidence that the proposed drug will be non-selective [41]. In our study, the most favourable docking result was determined for midostaurin with the S100A8 dimer with the estimated Ki as 68.48 nM and free energy of binding as-9.77 kcal/mol. There exists a strong logical correlation between inhibitor potency and selectivity [70] hence, more potent compounds are more selective because they can be applied at a lower dose.

## Conclusion

Our analysis suggests distinct transcriptomic signatures for KC with significantly high levels of S100A8 and EGFR expression involved in KC progression. Although further validation on larger dataset is needed to corroborate these findings, analysis of KC tissue is a promising tool for identifying biomarkers of interest. Protein-ligand interaction studies play a vital role in the structure based computational drug design. Our docking based findings shed insight into S100A8 protein as an attractive anticancer target and midostaurin as potential anticancer drug for therapeutic intervention in KC. S100A8 gained importance as target for anticancer drug development due to its central role in mediating inflammatory pathways that facilitate cancer metastasis. The docking simulatio of midostaurin with EGFR is also promising. Further investigations like quantitative structure-activity relationship (QSAR) studies are required to study and identify more favorable interactions with S100A8, EGFR and their partners. Furthermore, better binding ligands with higher affinity and efficacy can be designed and validated using combinatorial chemistry and co-crystallization approaches.

Therapeutic inhibition of kinase signaling cascades and emergence of novel clinically validated potent and selective kinase inhibitors facilitated by rational drug design is a proven boon especially for oncology. Studies to evaluate on- and off-target pharmacology (side effects) of these inhibitors in relation to efficacy and toxicity should be carried using KC cell lines. This will help in holistic monitoring of the changes in phosphorylation resulting from kinase inhibition.

## Supporting Information

S1 Table Differentially expressed genes of kidney cancer from Saudi patients; comparison of the genome-wide expression of kidney cancer with normal kidney tissue revealed 1478 differentially expressed genes, 852 up-regulated and 483 down-regulated, with ≥ 2 fold change and false discovery rate of p < 0.05. (XLS)

S2 Table Differentially expressed significant genes of kidney cancer from GEO retrieved data; expression profiling revealed 876, 1200 and 1258 differentially expressed genes for GSE781, GSE6344 and GSE7023 respectively, with ≥ 2 fold change and false discovery rate of p < 0.05. (XLS)
